# The *Salmonella* Effector SptP Dephosphorylates Host AAA+ ATPase VCP to Promote Development of its Intracellular Replicative Niche

**DOI:** 10.1016/j.chom.2009.01.010

**Published:** 2009-03-19

**Authors:** Daniel Humphreys, Peter J. Hume, Vassilis Koronakis

**Affiliations:** 1 Cambridge University Department of Pathology, Tennis Court Road, Cambridge CB2 1QP, UK

**Keywords:** MICROBIO, SIGNALING

## Abstract

Virulence effectors delivered into intestinal epithelial cells by *Salmonella* trigger actin remodeling to direct pathogen internalization and intracellular replication in *Salmonella*-containing vacuoles (SCVs). One such effector, SptP, functions early during pathogen entry to deactivate Rho GTPases and reverse pathogen-induced cytoskeletal changes following uptake. SptP also harbors a C-terminal protein tyrosine phosphatase (PTPase) domain with no clear host substrates. Investigating SptP's longevity in infected cells, we uncover a late function of SptP, showing that it associates with SCVs, and its PTPase activity increases pathogen replication. Direct SptP binding and specific dephosphorylation of the AAA+ ATPase valosin-containing protein (VCP/p97), a facilitator of cellular membrane fusion and protein degradation, enhanced pathogen replication in SCVs. VCP and its adaptors p47 and Ufd1 were necessary for generating *Salmonella*-induced filaments on SCVs, a membrane fusion event characteristic of the pathogen replicative phase. Thus, *Salmonella* regulates the biogenesis of an intracellular niche through SptP-mediated dephosphorylation of VCP.

## Introduction

To initiate infection of humans and animals, the intracellular pathogen *Salmonella enterica* serovar Typhimurium invades nonphagocytic intestinal cells, remodeling the host actin cytoskeleton and manipulating signal transduction pathways to induce membrane ruffling and trigger pathogen uptake by macropinocytosis. Internalized bacteria then multiply in specialized intracellular endosomal compartments called *Salmonella*-containing vacuoles (SCVs) (Cossart and Sansonetti, 2004). Although SCVs transiently display markers characteristic of early endosomes, e.g., Rab5 and transferrin receptor, they rapidly uncouple from the default endocytic pathway so that interaction with host lysosomes is limited and bacterial degradation is evaded (Garvis et al., 2001; Steele-Mortimer et al., 1999). The SCVs traffic to a Golgi-associated perinuclear position, where they intercept exocytic vesicles and fuse with late endosomes rich in LAMP1 (lysosomal-associated membrane protein 1), achieving an intracellular niche permissive for bacterial replication (Brumell et al., 2001; Kuhle et al., 2006; Meresse et al., 1999; Salcedo and Holden, 2003).

Both bacterial internalization and the subsequent SCV maturation are controlled by virulence effector proteins that are delivered into host cells via specialized type III secretion systems (T3SS) encoded by two *Salmonella* pathogenicity islands, SPI-1 and SPI-2 (Galan, 2001). While SPI-1-delivered effectors are translocated during the initial entry process, those delivered by SPI-2 are expressed later in SCVs. Nevertheless, it has become evident that several SPI-1-delivered effectors persist and influence postentry events (Brawn et al., 2007; Hernandez et al., 2004; Steele-Mortimer et al., 2002). We have reported that the SPI-1 actin-binding protein SipA, which promotes macropinocytosis and pathogen uptake (McGhie et al., 2001, 2004), localizes to SCVs, where it maintains the SCVs' perinuclear position (Brawn et al., 2007). The SPI-1 effector SopB also associates with vesicular membranes, where its phosphoinositide (PI) phosphatase activity leads to the accumulation of PI(3)P on the SCV and enhancement of membrane fusion with other PI(3)P-containing vesicles (Hernandez et al., 2004; Marcus et al., 2002). The SPI-1 effector SptP (*Salmonella* protein tyrosine phosphatase) has an N-terminal domain that functionally mimics GTPase-activating proteins (GAP) by deactivating the Rho GTPases Rac and Cdc42 and reversing the cytoskeletal rearrangements induced by the SPI-1 SopE/E2/SopB effectors to effect uptake (Fu and Galan, 1999; Patel and Galan, 2006). SptP translocation occurs during entry, where it downregulates membrane ruffling within 1 hr of pathogen internalization, but has also been shown to persist within host cells 3 hr after entry (Fu and Galan, 1999; Kubori and Galan, 2003).

We set out to investigate whether the apparent longevity of SptP in infected cells could allow additional intracellular activity subsequent to *Salmonella* internalization, possibly involving the SptP C-terminal protein tyrosine phosphatase (PTPase) domain (Kaniga et al., 1996; Stebbins and Galan, 2000), for which there are no clear host targets.

## Results

### SptP PTPase Activity Promotes *Salmonella* Intracellular Replication

**Figure 1:**
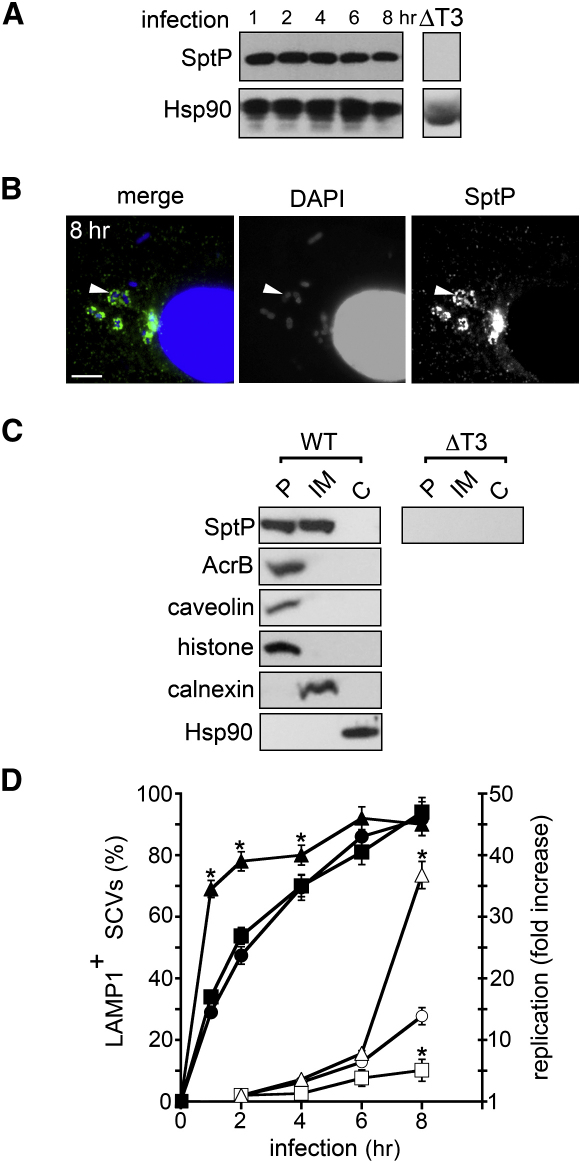
SptP Persistence and Promotion of Intracellular Replication (A) Persistence of SptP after cell infection. Lysates of HeLa cells infected with wild-type *S.* Typhimurium *sptP^FLAG^* were immunoblotted with antibodies against FLAG (SptP) and control Hsp90. No SptP^FLAG^ was detected following infection with T3SS-deficient *S.* Typhimurium Δ*invG* expressing SptP^FLAG^ (ΔT3). (B) Intracellular localization of SptP. HeLa cells infected as in (A) were stained with DAPI (host nuclei and bacteria; blue) and antibodies against FLAG (SptP; green). Scale bars, 5 μm. (C) Subcellular localization of SptP. HeLa cells infected as in (A) were fractionated into the pellet (P), internal membranes (IM), and host cytoplasm (C). Samples were immunoblotted with antibodies against FLAG (SptP), AcrB, caveolin, histone, calnexin, and Hsp90. No SptP^FLAG^ was detected following infection with T3SS-deficient *S.* Typhimurium *ΔinvG* expressing SptP^FLAG^ (ΔT3). (D) SptP influence on LAMP1 acquisition by SCVs (closed symbols) and intracellular *S.* Typhimurium replication (open symbols). HeLa cells were infected in parallel with WT (circles), Δ*sptP* (squares), and Δ*sptP* pSPTP (triangles) strains. Infected cells were stained with DAPI and antibodies against LAMP1 at indicated times postinfection, and LAMP1-positive SCVs were quantified (left axis). In parallel, intracellular replication (right axis) was measured. Following infection (0 hr), gentamicin was added at 1 hr to kill extracellular bacteria, and replication was quantified by colony counts at indicated times. Data points are shown as geometric means ± 95% confidence intervals. Asterisks indicate a significant difference from wild-type (p < 0.05, ANOVA; n ⩾ 3).

To track SptP after bacterial internalization, we generated a strain of wild-type *S.* Typhimurium, ATCC 14028, in which the chromosomal *sptP* gene was C-terminally fused to nucleotides encoding a 3×FLAG epitope tag (*sptP^FLAG^*) to enhance detection (Experimental Procedures). HeLa cell infections were initiated by incubation with this strain for 1 hr, after which extracellular bacteria were killed by adding the antibiotic gentamicin, which does not penetrate HeLa cells. Host cells were lysed at intervals up to 8 hr postinfection, and T3SS-delivered SptP^FLAG^ was detected at all time points by immunoblotting (Figure 1A). This observation confirmed and extended data (Kubori and Galan, 2003) showing that SptP persists a long time after bacterial internalization by avoiding proteasome-dependent degradation (Figure S1A). Immunofluorescence of the infected HeLa cells indicated that SptP^FLAG^ accumulated around 15% ± 3% of intracellular bacteria, as exemplified at the 8 hr point in Figure 1B. Further immunofluorescence microscopy showed that SptP-positive bacteria were found to reside within SCVs (Figure S1B). To localize T3SS-delivered SptP^FLAG^ with more precision, we mechanically fractionated HeLa cells 8 hr postinfection and verified separation of the pellet containing plasma membranes (marked by caveolin), nuclei (histone), and bacteria (AcrB) from internal membranes (calnexin) and cytoplasm (Hsp90) (Figure 1C). No SptP^FLAG^ was observed in the host cytoplasm, but it was clearly present in the pellet fraction containing plasma membranes, nuclei, and internalized bacteria and also the fraction containing internal membranes (Figure 1C). The same pattern of localization was evident at the earlier time points (data not shown), showing that the persistent SptP is present at cellular membranes when bacteria reside within SCVs.

It is accepted that soon after formation, SCVs fuse with vesicular membranes enriched in the late endosomal or lysosomal marker LAMP1, and from 4–6 hr after internalization, the start of intracellular replication is marked by generation of tubulovesicular *Salmonella*-induced filaments (Sifs) (Steele-Mortimer, 2008). To assess whether SptP^FLAG^ might influence these postinvasion events, HeLa cells were infected in parallel by wild-type *Salmonella*, an *sptP* chromosomal deletion mutant (*ΔsptP*), and the mutant complemented with a recombinant *sptP* gene on a plasmid (*ΔsptP* pSPTP, which translocates 50% more SptP^FLAG^ than wild-type bacteria [Figure S2A] [Cain et al., 2004]). Infected cells were assayed for LAMP1 acquisition by SCVs, for Sif formation, and for intracellular replication. Deletion of *sptP* did not alter LAMP1 acquisition from that induced by the wild-type *S.* Typhimurium (Figure 1D, closed squares versus circles), but complementing the mutant with pSPTP accelerated LAMP1 recruitment during the first 4 hr postinfection (Figure 1D, closed triangles). At 6 hr, Sif formation was clearly visible in 54.6% ± 1.1% of wild-type infected cells (Figure S2B). Although SptP was apparently excluded from Sif structures (Figure 1B), their formation was reduced to 26.4% ± 1.1% with the *ΔsptP* mutant and was restored to wild-type levels by complementing with pSPTP. This was mirrored in intracellular replication assayed at 8 hr (Figure 1D, open squares versus circles), which was reduced from 14.3- ± 1.4-fold to 4.2- ± 2.6-fold with the *ΔsptP* mutant, but was recovered—indeed, enhanced—to 35.2- ± 2.8-fold when *ΔsptP* was complemented with pSPTP (Figure 1D, open triangles versus circles). Moreover, in each case, intracellular replication at the 8 hr point occurred within intact LAMP1-positive SCVs (Figure 1D, closed symbols).

To determine which domain of SptP is responsible for the promotion of intracellular replication and Sif generation, amino acid substitutions were introduced into the catalytic sites of the N-terminal GAP (R209A) or C-terminal PTPase (D441A) domains to specifically impair their enzymatic activity (Fu and Galan, 1999; Murli et al., 2001), and the corresponding expression plasmids were introduced into *ΔsptP*. FLAG-tagged SptP^R209A^ and SptP^D441A^ were associated with the pellet and internal membranes (Figure S2C), as was wild-type SptP, shown in Figure 1C, but while both strains resided within SCVs during cell infection, only SptP^R209A^ restored Sif formation and promoted intracellular replication (Figure S2B).

### SptP Binds and Dephosphorylates the Host AAA+ ATPase VCP

**Figure 2:**
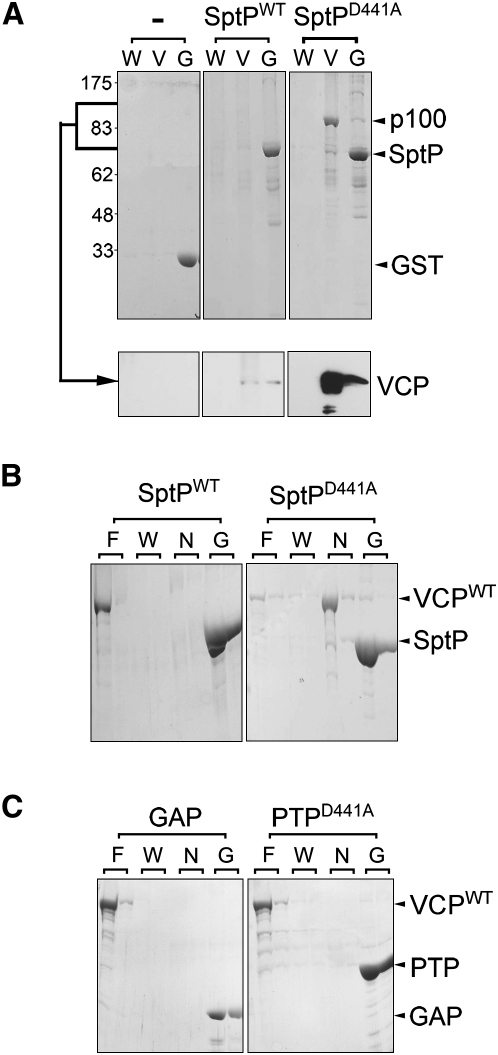
SptP Interaction with the AAA+ ATPase VCP (A) Isolation of a stabilized SptP substrate intermediate from porcine brain extract. Purified GST control (−), GST-SptP (SptP^WT^), and GST-SptP^D441A^ (SptP^D441A^) were immobilized on glutathione-Sepharose beads and incubated with brain extract. Beads were washed (W) before eluting bound eukaryotic proteins with 1 mM vanadate (V). Immobilized SptP and remaining proteins were eluted with reduced glutathione (G). Eluted fractions were analyzed by Coomassie blue-stained SDS-PAGE (upper panels) with molecular weight markers in kDa (left), and then immunoblotted (lower panels) using antibodies against VCP (arrowed). (B) Interaction between purified recombinant SptP and VCP. SptP^WT^ and SptP^D441A^ were immobilized on glutathione Sepharose beads and incubated with His-tagged VCP (VCP). Also shown are flowthrough containing unbound protein (F; lanes 1 and 2), washes (W; lanes 3 and 4), and proteins eluted with 1 M NaCl (N; lanes 5 and 6). Immobilized SptP and remaining VCP were eluted with reduced glutathione (G; lanes 7 and 8). Fractions were analyzed by Coomassie blue-stained SDS-PAGE. (C) Interaction between purified recombinant GST-GAP (GAP) or GST-PTP^D441A^ (PTP^D441A^) SptP domains with VCP, performed as in (B).

The cellular targets of the SptP PTPase are unclear. Substrate associations with active PTPases are transient and difficult to identify (Tiganis and Bennett, 2007). Nevertheless, disabling the active site of PTPases stalls catalysis and can stabilize target binding to allow the capture of trapped substrates (Flint et al., 1997). SptP carrying the aspartate-to-alanine substitution at residue 441 was fused to GST, immobilized onto glutathione Sepharose beads, and used to probe porcine brain extract for potential host targets. Following parallel incubation of immobilized GST-SptP^D441A^ (SptP^D441A^) and wild-type GST-SptP (SptP^WT^) with brain extract (Figure 2A, upper), the beads were washed (W) and mammalian proteins eluted with extract buffer containing vanadate (V), a phosphotyrosine mimic that competitively inhibits PTPase-substrate binding (Huyer et al., 1997). Remaining SptP was then eluted with extract buffer containing reduced glutathione (G). While SptP^WT^ did not isolate any host protein discernable by Coomassie blue-stained SDS-PAGE, SptP^D441A^ captured a protein migrating at approximately 100 kDa (Figure 2A, upper p100). Capture of this protein by SptP^D441A^ was blocked by preincubating the brain extract with vanadate prior to addition of the beads (data not shown). The putative p100 target was isolated and identified, using mass spectrometry, as VCP (valosin-containing protein, also named p97), a eukaryotic ATPase associated with a variety of activities (AAA+ family) that facilitates disassembly of protein complexes during membrane fusion and protein degradation (Ye, 2006). Immunoblotting the same fractions from the pull-down experiments with antisera against VCP (Figure 2A, lower) reconfirmed that the SptP^D441A^-bound protein was VCP, and also indicated a weaker interaction between VCP and SptP^WT^. When recombinant His-tagged VCP (VCP^WT^) was generated and incubated in binding buffer with immobilized SptP^D441A^ (Figure 2B), direct interaction was evident and could be disrupted with 1 M NaCl (N), demonstrating that binding requires no additional factors and probably involves charge-charge associations. To ascertain whether the SptP PTPase domain alone is sufficient for VCP binding, the GAP (residues 162–290) and PTPase^D441A^ (residues 300–542) domains were fused independently to GST and purified. In agreement with published findings (Fu and Galan, 1998), both GST-GAP and GST-PTPase domains retained their enzymatic activity (data not shown), but neither SptP GAP or PTPase^D441A^ was able to bind recombinant VCP^WT^, as shown in empty lanes (N) in Figure 2C, or endogenous porcine VCP (data not shown).

**Figure 3:**
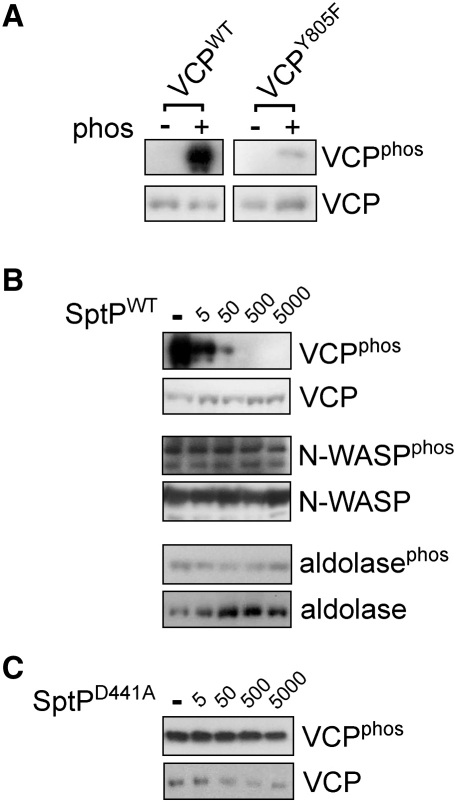
SptP PTPase Activity toward VCP In Vitro (A) In vitro phosphorylation of VCP at tyrosine 805. Purified recombinant wild-type VCP (VCP^WT^) and VCP with tyrosine 805 substituted for phenylalanine (VCP^Y805F^) were phosphorylated in vitro (phos; −/+), and phosphorylation was assessed by immunoblotting with antibodies against phosphotyrosine (VCP^phos^) and VCP. (B) SptP PTPase activity toward VCP. In vitro phosphorylated recombinant VCP, N-WASP, and aldolase (2 μM) were incubated with picomolar concentrations (top) of SptP^WT^. Phosphorylation was assessed by immunoblotting samples with antibodies against phosphotyrosine (VCP^phos^, N-WASP^phos^, and aldolase^phos^) and VCP, N-WASP, and aldolase as controls. (C) SptP^D441A^ PTPase activity toward VCP, performed as in (A).

To assess further whether VCP is an SptP PTPase substrate, purified VCP^WT^ was first tyrosine phosphorylated in vitro (Figure 3A; VCP^phos^). The major site of phosphorylation in VCP is known to be tyrosine 805 (Egerton and Samelson, 1994; Zhao et al., 2007), and VCP phosphorylation at this functionally relevant tyrosine was confirmed by marked reduction in phosphorylation when tyrosine 805 was substituted for phenylalanine to generate VCP^Y805F^ (Figure 3A; VCP^phos^). When phosphorylated VCP^WT^ (2 μM) was incubated with even low picomolar concentrations of SptP^WT^, it was dephosphorylated, while phosphorylation of control proteins N-WASP and aldolase was unaffected, even by nanomolar concentrations of SptP^WT^ (Figure 3B; VCP^phos^, N-WASP^phos^, aldolase^phos^). No dephosphorylation of phosphorylated VCP^WT^ was evident when incubated with SptP^D441A^ (Figure 3C) or individual SptP GAP and PTPase domains (data not shown).

### The SptP Substrate VCP Promotes Intracellular Replication of *Salmonella*

**Figure 4:**
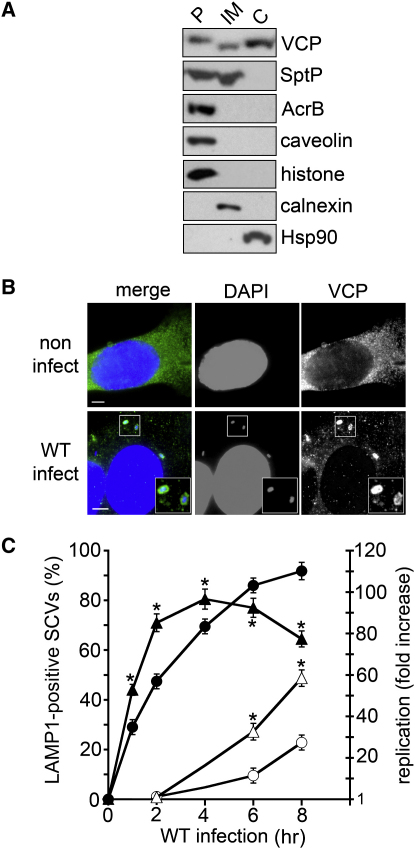
VCP Promotion of *S.* Typhimurium Intracellular Replication (A) Subcellular localization of VCP and SptP^FLAG^ after cell infection. HeLa cells 4 hr postinfection with wild-type *S.* Typhimurium were mechanically fractionated into the pellet (P), internal membranes (IM), and host cytoplasm (C) before immunoblotting with antibodies against VCP, FLAG (SptP), AcrB, caveolin, histone, calnexin, and Hsp90. (B) Localization of VCP during wild-type *S.* Typhimurium cell infection. Noninfected and infected (WT) HeLa cells 4 hr postinfection were stained with DAPI (host nuclei and bacteria; blue) and antibodies against VCP (green). Inset shows VCP localization around bacteria. Scale bars, 5 μm. (C) Effect of VCP siRNA on LAMP1 acquisition by SCVs (closed symbols) and *S.* Typhimurium intracellular replication (open symbols). HeLa cells treated with VCP siRNA (triangles) or control siRNA (circles) were infected with wild-type *S.* Typhimurium. Infected cells were then stained with DAPI and antibodies against LAMP1, and LAMP1-positive SCVs were quantified (left axis). In parallel, intracellular replication (right axis) was measured. Following infection (0 hr), gentamicin was added at 1 hr to kill extracellular bacteria, and replication was quantified by colony counts at indicated times. Data points are shown as geometric means ± 95% confidence intervals. Asterisks indicate a significant difference from wild-type (p < 0.05, ANOVA; n ⩾ 3).

Our data indicate that the SptP PTPase activity promotes Sif formation and intracellular *Salmonella* replication, and that it can specifically dephosphorylate VCP in vitro. We therefore investigated whether the host VCP influences intracellular replication. We mechanically fractionated HeLa cells infected with wild-type *S.* Typhimurium, as in Figure 1C, and found (Figure 4A) that VCP was present in the pellet fraction containing plasma membranes, nuclei, and internalized bacteria, as well as in those containing internal membranes and those containing cytoplasm (the same pattern was seen in noninfected control HeLa cells [data not shown]). Parallel immunofluorescence, as exemplified in the inset boxes (Figure 4B), indicated that VCP localized to approximately 12% ± 4% of intracellular bacteria up to 8 hr postinfection. The low percentage of VCP-positive intracellular bacteria was similar to that seen for SptP (Figure 1B); however, no colocalization was observed.

To examine further the role of VCP during cell infection with wild-type *S.* Typhimurium, we targeted VCP mRNA for degradation in HeLa cells by transfecting with VCP siRNA. VCP depletion was confirmed by immunoblotting (Figure S3A), and no reduction in bacterial internalization was observed in the VCP-depleted HeLa cells (data not shown). However, as when HeLa cells were infected with *ΔsptP* expressing pSPTP (Figure 1B), LAMP1 recruitment by SCVs was accelerated in the VCP-depleted cells (Figure 4C, closed symbols). From 4 hr, the number of LAMP1-positive SCVs declined in VCP-depleted cells, revealing loss of SCV integrity and bacterial release into the nutrient-rich HeLa cell cytosol, where it is accepted that *Salmonella* hyperreplicate (Brumell et al., 2002; Perrin et al., 2004). Indeed, SCV lysis following VCP-depletion coincided with increased intracellular replication (Figure 4C, open symbols) and a marked reduction in Sif formation from 48% to 6% in Figure S3B. Increased intracellular replication at 8 hr following VCP depletion was reconfirmed with distinct nonoverlapping siRNAs against VCP (data not shown).

VCP is necessary for diverse ubiquitin-dependent processes in host cells. Our VCP RNAi data could indicate that VCP is required for maintaining SCV integrity. Alternatively, VCP depletion may create a poor host for *Salmonella* replication in SCVs. VCP function relies upon its association with at least two sets of major pathway-specific adaptor proteins (Ye, 2006). The heterodimeric adaptors Npl4 and Ufd1 associate with VCP during protein degradation, while VCP controls membrane fusion events via its adaptor, p47. To further examine the function of VCP during intracellular replication of wild-type *S.* Typhimurium, we depleted HeLa cells of Npl4, Ufd1, or p47 by siRNA transfection (Figure S3C), then assayed for LAMP1 acquisition, Sif formation, and intracellular replication (Table 1). Npl4, Ufd1, and p47 depletion did not affect the cellular levels of VCP (Figure S3D) nor LAMP1 acquisition by wild-type SCVs, and bacteria remained within intact SCVs for up to 8 hr (data not shown). No statistically significant differences in Sif formation at 6 hr were observed in Npl4-depleted cells, while Ufd1 depletion reduced Sif formation from 58.2% ± 1.1% in control cells to 38.2% ± 1.2%, and in p47-depleted cells, Sif formation was retarded further to 26.7% ± 1.3% (Table 1). As observed with the *ΔsptP* mutant (Figure 1B), the defect in Sif formation by wild-type *S.* Typhimurium was mirrored in intracellular replication at 8 hr (Table 1), which was reduced from 29.6- ± 1.0-fold in control cells to 14.9- ± 2.2-fold and 7.6- ± 1.2-fold in Ufd1- and p47-depleted cells, respectively.

### SptP Dephosphorylates VCP during *Salmonella* Cell Infection

**Figure 5:**
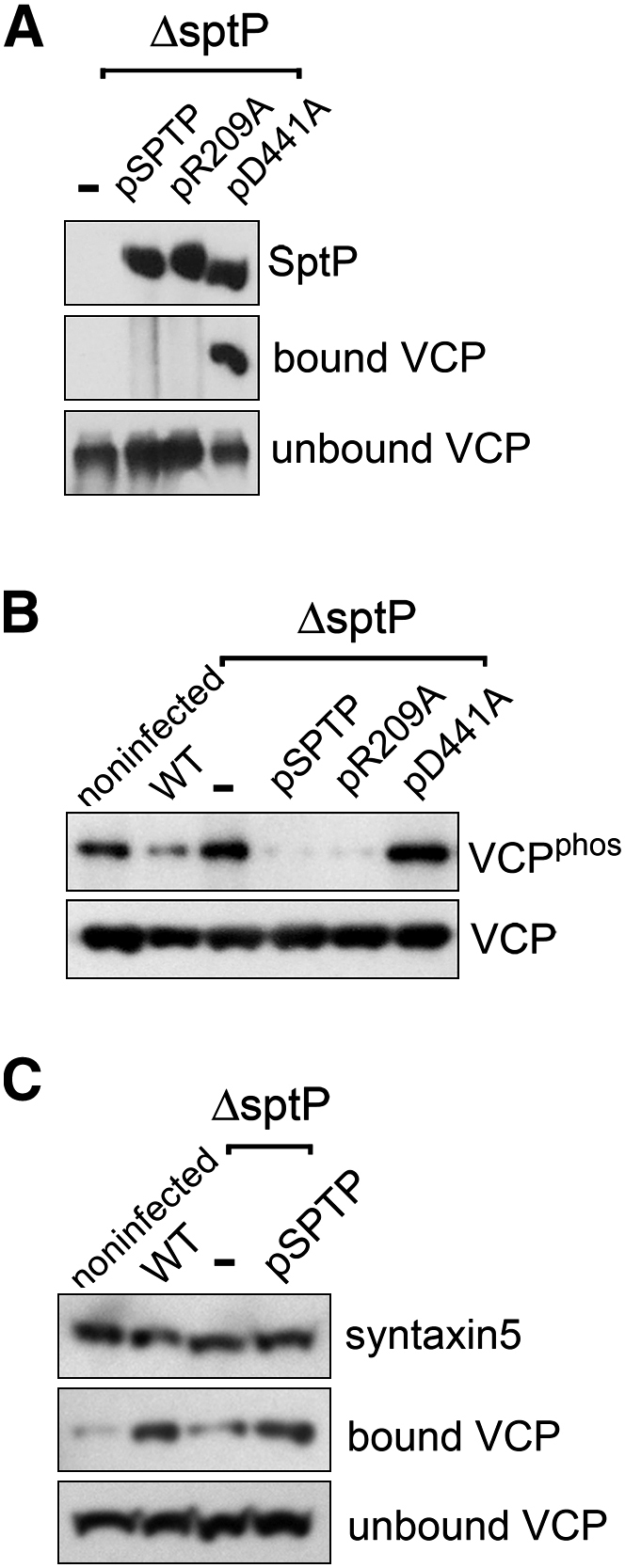
SptP-Dependent VCP Dephosphorylation during Infection (A) SptP interaction with VCP. HeLa cells were infected with *S.* Typhimurium *ΔsptP* expressing plasmid-encoded FLAG-tagged SptP variants (−, pSPTP, pR209A, pD441A). At 4 hr postinfection, cells were lysed with detergent, and SptP variants were immunoprecipitated from cell lysates with antibodies against SptP immobilized on protein G Sepharose. Eluted immunoprecipitates were assayed for presence of SptP and bound VCP and also supernatants for unbound VCP by immunoblotting with antibodies against FLAG (SptP) and VCP. (B) SptP-dependent dephosphorylation of VCP during *S.* Typhimurium cell infection. HeLa cells were individually infected with *S.* Typhimurium strains, described in (A), and wild-type (WT). At 4 hr postinfection, cells were lysed with detergent, and VCP was immunoprecipitated using antibodies against VCP immobilized on protein G Sepharose. Phosphorylation status of immunoprecipitated VCP was assessed by immunoblotting with antibodies against phosphotyrosine (VCP^phos^) and VCP serving as a loading control (VCP). (C) SptP-dependent syntaxin5 interaction with VCP during cell infection. HeLa cells transiently transfected with pcDNA3.1-HA-STX5 were either noninfected or infected in parallel with wild-type *S.* Typhimurium, the *ΔsptP* (−) null mutant, or *ΔsptP* expressing plasmid-encoded SptP (pSPTP). HA-tagged syntaxin5 was immunoprecipitated from cell lysates with antibodies against HA immobilized on protein G Sepharose. Eluted immunoprecipitates were assayed for presence of syntaxin5 and bound VCP and also supernatants for unbound VCP by immunoblotting with antibodies against HA (syntaxin5) and VCP.

Next, we assessed the possibility that SptP delivered into mammalian cells specifically targets VCP. HeLa cells were infected in parallel with the *ΔsptP* mutant or with the same mutant expressing the FLAG-tagged SptP variants listed in Figure S2B. After 4 hr, cells were lysed with detergent and SptP isolated by immunoprecipitation (Figure 5A). Consistent with our earlier in vitro binding data (Figures 2A and 2B), VCP was coprecipitated (bound) by immobilized SptP^D441A^ lacking PTPase activity, but not equivalently tagged SptP^WT^ or GAP-defective SptP^R209A^, where VCP was only present in unbound fractions (Figure 5A). Parallel immunofluorescence revealed that, unlike SptP^WT^, the substrate-trap SptP^D441A^ was absent from SCVs and localized diffusely throughout the perinuclear region (Figure S4A), implying that SptP may target VCP at membranes distinct from the SCV. Other persistent (FLAG-tagged) SPI-1 effectors, SopB and SipA (Brawn et al., 2007; Hernandez et al., 2004), did not bind VCP (Figure S4B).

To demonstrate SptP-dependent VCP dephosphorylation in these cells, VCP was immunoprecipitated from cell lysates and assessed for tyrosine phosphorylation by immunoblotting with antibodies against phosphotyrosine (Figure 5B). Phosphorylated VCP was evident in noninfected control cells (Figure 5B; VCP^phos^), but there was a clear reduction in the phosphorylated form in cells infected with wild-type *S.* Typhimurium. This reduction was not apparent when cells were infected with the *ΔsptP* null mutant (−). No significant SptP-dependent changes in global cellular phosphotyrosine levels were observed, indicating specificity for VCP (Figure S4C). When HeLa cells were infected with *S.* Typhimurium *ΔsptP* delivering augmented levels of plasmid-encoded SptP (pSPTP) (Figure 5B), dephosphorylation of VCP was enhanced substantially over that of cells infected with the wild-type *S.* Typhimurium, and this was also true following HeLa cell infection with *S.* Typhimurium *ΔsptP* delivering SptP^R209A^ (pR209A). In contrast, the PTPase mutant SptP^D441A^ (pD441A) was unable to dephosphorylate VCP.

The major adaptor proteins (p47/Ufd1/Npl4) broadly determine VCP function, with the precise pathway specified by interaction with further specific coadaptors and/or substrates that can be controlled through dephosphorylation of VCP (Lavoie et al., 2000; Zhao et al., 2007). For example, VCP-p47 complexes associate with the t-SNARE syntaxin5, which is a VCP adaptor involved in membrane fusion events (Rabouille et al., 1998). The association of VCP-p47 with syntaxin5 is known to be promoted by dephosphorylation of VCP, and this leads to increased membrane fusion events (Lavoie et al., 2000). Therefore, we examined whether SptP-dependent dephosphorylation of VCP promotes associations with syntaxin5 during HeLa cell infection. First, HeLa cells were transiently transfected with a mammalian expression vector encoding HA-tagged syntaxin5, which was immunoprecipitated from cell lysates and assessed for coprecipitated VCP by immunoblotting (Figure 5C). In all cases, VCP was predominantly observed in the unbound supernatant fraction, but VCP was also coprecipitated by immobilized syntaxin5 from noninfected controls cells (bound VCP). There was a clear enhancement in the amount of bound VCP in cells infected with wild-type *S*. Typhimurium, whereas this increase was not evident when cells were infected with the *ΔsptP* null mutant (−). When HeLa cells were infected with the *ΔsptP* strain delivering plasmid-encoded SptP (pSPTP), the levels of bound VCP were enhanced considerably over that isolated from noninfected control cells and those infected with the *ΔsptP* null mutant alone.

## Discussion

In this study, we reveal a late function for the *Salmonella* SPI-1 effector SptP. We show that this effector persisted for at least 8 hr after *Salmonella* internalization into HeLa cells, and that like the other late-acting effectors, SopB and SipA, it was located at internal membranes, including SCVs. Our initial data indicated that, in infected cells, the SptP PTPase activity promotes bacterial replication in SCVs, so we set out to identify the putative host cellular substrate(s) of the SptP PTPase. By using the D441A variant of SptP, in which the PTPase site was specifically disabled, we were able to trap a single substrate and identify it as VCP, a host AAA+ ATPase that performs a motor-like function to facilitate protein complex disassembly in diverse cellular processes (Ye, 2006).

We showed that SptP-VCP interaction in vitro is direct, requiring no third protein, but the SptP PTPase domain alone was not sufficient to enable VCP binding and in vitro dephosphorylation. This requirement for both SptP effector domains could explain why VCP was not previously identified in a yeast two-hybrid screen using only the isolated SptP PTPase domain (this work indicated the intermediate filament protein vimentin as a potential binding partner for SptP, but the significance of this remains unclear [Murli et al., 2001]). The dual activities of SptP contrast to the situation in *Yersinia*, which has two distinct SptP homologs; the Rho GAP mimic YopE acts on Rho GTPases (Black and Bliska, 2000), while the PTPase YopH targets tyrosine-phosphorylated proteins at focal adhesion complexes (Black and Bliska, 1997). Our data indicate that fusion of the GAP and PTPase moieties is required for SptP-dependent VCP dephosphorylation, compatible with evidence from many mammalian tyrosine phosphatases that employ noncatalytic domains to mediate substrate interactions (Tiganis and Bennett, 2007), and is presumably responsible for the observed specificity of SptP. Consistent with this hypothesis, GAP-defective SptP^R209A^ binds and dephosphorylates VCP in vitro (Figure S5).

We were able to confirm that SptP specifically dephosphorylates host VCP during cell infection, and this did not occur when the PTPase active site was disabled. This dephosphorylation event appears to promote *Salmonella* infection (Figure S6A). Depleting the phosphorylated form of VCP through augmenting delivered SptP levels enhanced pathogen replication in SCVs, whereas replication of the *ΔsptP* strain, which is unable to dephosphorylate VCP, is impaired (Figure S6B). We found that RNAi depletion of VCP increased intracellular *Salmonella* replication. However, unlike the increase seen when SptP delivery was augmented, this was due to induced SCV instability and consequent bacterial release into the nutrient-rich cytoplasm (Figure S6C). This is not entirely surprising, as VCP depletion impairs multiple cellular processes (Wojcik et al., 2004). To probe the role of VCP in SCV biogenesis more precisely, we additionally targeted the major pathway-specific adaptors through which VCP effects its myriad functions (Dreveny et al., 2004; Ye, 2006). VCP promotion of Sif formation and intracellular wild-type *Salmonella* replication was impaired by knockdown of either the membrane fusion adaptor p47 or the protein degradation adaptor Ufd1 (Figure S6D), as observed following infection with the *ΔsptP* strain. Although Npl4 forms a heterodimeric complex with Ufd1, we observed no effect following Npl4 depletion. This may be due to the relative inefficiency of the Npl4 RNAi knockdown. Interestingly, the VCP-Ufd1-Npl4 pathway has been shown to be required for replication of the intracellular pathogen *Legionella pneumophila* (Dorer et al., 2006). We show that *Salmonella* exploits both p47- and Ufd1-dependent pathways to promote SCV biogenesis.

How does dephosphorylation affect VCP function? The distribution of VCP, including its recruitment to the SCV, was found to be independent of SptP (data not shown), suggesting that dephosphorylation does not influence localization. However, the sheer abundance of VCP (up to 1% of total cellular protein) means we cannot rule out subtle changes in the distribution of small subpopulations. A low proportion of SCVs localized with VCP, as was also the case for SptP, indicating that interactions with the SCV are likely transient or, alternatively, that the site of action of VCP is not the SCV itself. Indeed, we identified a significant pool of both VCP and SptP on other internal membranes that may interact with the SCV during its maturation. In addition, the substrate-trap SptP^D441A^ was absent from SCVs (Figure S4A), implying that SptP may target VCP at membranes distinct from the SCV.

**Figure 6:**
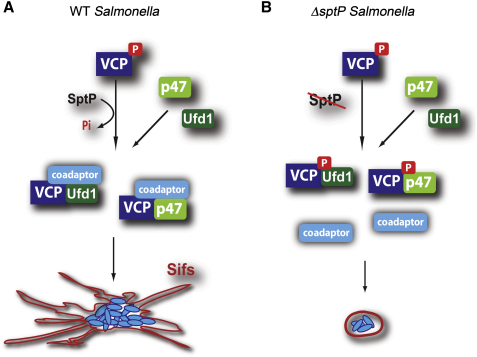
Mechanism for SptP Promotion of Intracellular Replication (A) SptP PTPase activity promotes intracellular replication. SptP-dependent dephosphorylation of VCP allows it to promote Sif formation and intracellular replication through the major adaptors p47 and Ufd1. SptP-dependent dephosphorylation of VCP enhances its association with pathway-specific coadaptors to determine downstream functions. (B) *sptP* null *Salmonella* are retarded for intracellular replication. Internalized *ΔsptP Salmonella* reside in SCVs, but VCP is not dephosphorylated. This prevents the association of VCP-p47 and VCP-Ufd1 complexes with pathway-specific coadaptor proteins and thus impairs specific VCP functions required for promoting Sif formation and intracellular replication.

The activities of pathway-specific VCP-adaptor complexes are further defined by interaction with specific coadaptors and/or substrates in a hierarchical manner (Dreveny et al., 2004; Schuberth and Buchberger, 2008). Over forty proteins are currently known to bind VCP, and the molecular details of these interactions are only beginning to emerge (Yeung et al., 2008). Several VCP adaptors and coadaptors whose binding is prevented by VCP phosphorylation (Li et al., 2008; Madsen et al., 2008; Zhao et al., 2007) have, however, been identified. Both p47 and Ufd1-Npl4 have been shown to bind to N-terminal regions of VCP, distal to the site of tyrosine phosphorylation (Pye et al., 2007). However, the binding of VCP-p47 and VCP-Ufd1-Npl4 complexes to various coadaptors can involve the C-terminal phosphotyrosine (Madsen et al., 2008). One such example is the interaction of VCP-p47 with the t-SNARE syntaxin5, which is known to be promoted by dephosphorylation of VCP and leads to increased membrane fusion events (Lavoie et al., 2000). SptP may therefore manipulate VCP function by regulating its interaction with specific coadaptors. Indeed, we show that during infection, SptP-mediated dephosphorylation leads to an increase in the binding of VCP to syntaxin5. In the absence of SptP, this interaction was greatly reduced. By enhancing VCP-syntaxin5 association, SptP thus promotes fusion events within the cell that are likely crucial for the maturation and maintenance of the SCV (Figure 6). This is consistent with the accelerated acquisition of LAMP1 observed in strains delivering augmented levels of SptP. Further work is required to determine whether any other of the plethora of VCP-interacting proteins are also regulated by *Salmonella* in this manner. Our findings uncover a target for the enigmatic SptP PTPase domain that reveals a mechanism by which *Salmonella* subverts host processes to promote the biogenesis of its intracellular niche.

## Experimental Procedures

### Bacterial Strains and Plasmids

The *ΔsptP* and *ΔinvG sptP::3×FLAG* mutants and wild-type *sptP::3×FLAG*, *sopE2::3×FLAG*, and *sopB::3×FLAG* were constructed using λ Red recombination as described for *sipA::3×FLAG* (Brawn et al., 2007), and the bacterial expression plasmid pSPTP has been described (Cain et al., 2004). The FLAG epitope tag confers no intrinsic membrane affinity to cytosolic proteins and has been shown to enhance effector protein detection (Cain et al., 2004). To construct GST fusions, PCR products corresponding to *sptP* (base pairs 486–1629), *sptP* GAP domain (base pairs 486–870), and *sptP* PTPase domain (base pairs 900–1629) were inserted into the BamHI*/*EcoRI sites of pGEX2T. To construct the His-tagged VCP fusion, full-length rat *vcp* was amplified from a cDNA template (accession number NM_007126; obtained from RZPD, Germany) and inserted into the NdeI/BamHI sites of pET15b (pHIS-VCP). To construct the HA epitope-tagged syntaxin5 fusion, full-length human *syntaxin5* was amplified from a cDNA template (pOTB7-syntaxin5, MRC geneservice, accession number NP_003155) in frame with sequence corresponding to an N-terminal HA epitope tag and products inserted into the HindIII/XbaI sites of pcDNA3.1+. Point mutations were introduced into *sptP* and *vcp* by site-directed mutagenesis (Stratagene QuickChange Site-Directed Mutagenesis Kit). GST and His-tagged proteins were purified as described (Cain et al., 2004). His-tagged aldolase and N-WASP were gifts from Doctor E.J. McGhie.

### Mammalian Cell Culture and RNA Interference

Mammalian HeLa cells (ATCC-CCL-2) were routinely cultured in complete growth media consisting of DMEM supplemented with 10% (v/v) fetal calf serum, 2 mM L-glutamine, 200 μg/ml^−1^ streptomycin, and 100 Uml^−1^ penicillin (37°C, 5% CO_2_). For RNA interference, siRNA from QIAGEN against VCP (gene accession number NM_007126, Hs_VCP_7 Seq: AACAGCCATTCTCAAACAGAA, Hs_VCP_6 Seq: AAGATGGATCTCATTGACCTA, Hs_VCP_8 Seq: ACCCTGATTGCTCGAGCTGTA), p47 (gene accession number NM_016143, Hs_NSFL1C_1/5 Seq: CACCAGCATGTTGTACGGAAA, ATCGTGCAGCGGTTAACATAA), Npl4 (gene accession number NM_017921, Hs_NPLOC4_1/2 Seq: CACGCCTAATCCTGTTGACAA, CCGAAAGGGTACCGTCCGCTA), Ufd1 (gene accession number NM_001035247, Hs_UFD1L_5/6 Seq: CTGGGCTACAAAGAACCCGAA, CACTGGATGATGCAGAACTTA), or Allstars negative control siRNA (QIAGEN) was transfected into HeLa cells with oligofectamine transfection reagent (Invitrogen) according to manufacturer's instructions. Transfection mixture was replaced after 8–24 hr with complete growth medium and cells cultured 72 hr in total. RNAi efficiency was determined by immunoblotting or QuantiTect SYBR Green qRT-PCR according to manufacturer's instructions (QIAGEN).

### Infection and Mechanical Fractionation of HeLa Cells

The ability of *Salmonella* strains to invade and replicate in cultured HeLa cells was assessed by gentamicin protection. When appropriate, infected cells were processed for immunofluorescence microscopy, and images were assembled as described (Brawn et al., 2007). For mechanical fractionation, infected HeLa cells were disrupted by passage through a 22G needle in lysis buffer containing 10 mM HEPES (pH 7.4), 250 mM sucrose, 0.5 mM EDTA, and Complete EDTA-free protease inhibitor cocktail (Roche). Low-speed centrifugation (3000 *g*, 10 min) pelleted plasma membranes and internalized bacteria, and ultracentrifugation (180,000 *g*, 20 min) separated internal membranes from cytoplasm.

### Statistical Analysis

All experiments were repeated at least three times. Geometric means were calculated and significance determined by one-way ANOVA followed by a post hoc Dunnett's comparison. p < 0.05 was considered significant.

### In Vitro Pull-Down

GST fusion proteins, bound to glutathione Sepharose 4B beads (GE Healthcare; Little Chalfont, UK) (4°C, 2 hr) in extract buffer (100 mM MES [pH 6.8], 5 mM MgCl_2_, 0.5 mM EDTA, 1 mM EGTA with 0.5 mM ATP, 1 mM GTP, 0.5 mM DTT, and Complete EDTA-free protease inhibitor cocktail [Roche]), were incubated with porcine brain extract (RT, 10 min). Beads were immobilized on a column then washed with extract buffer (50 ml). Bound proteins were eluted by addition of extract buffer supplemented with 1 mM vanadate followed by extract buffer supplemented with 10 mM reduced glutathione. For in vitro binding experiments, GST fusion proteins bound to glutathione Sepharose 4B beads in binding buffer (20 mM Tris-HCL [pH 7.4], 150 mM NaCl, 1 mM MgCl_2_, 0.5 mM ATP, 1 mM GTP, and 0.5 mM DTT) were incubated with equimolar amounts of His-tagged VCP (RT, 10 min). The mixture was immobilized and washed with binding buffer (10 ml), and fractions were collected. Bound proteins were eluted by addition of extract buffer supplemented with 1 M NaCl followed by extract buffer supplemented with 10 mM reduced glutathione. Proteins were extracted from SDS-PAGE gels and identified by electrospray ionization LC-MS (Cambridge Centre for Proteomics, University of Cambridge, UK).

### Immunoblotting and Antibodies

Primary antibodies for immunoblotting, immunofluorescence, and immunoprecipitation were rabbit polyclonal anti-SptP (Cain et al., 2004), purified with GST-SptP covalently bound to amino link coupling gel according to manufacturer's instructions (Pierce; Rockford, IL); mouse monoclonal anti-VCP (Abcam; Cambridge, MA); mouse monoclonal anti-p97 (Research Diagnostics; Concord, MA); rabbit anti-p47 (HME22, gift from Doctor Hemmo Myer); mouse monoclonal anti-Ufd1 (5E2, gift from Doctor Hemmo Myer); mouse monoclonal anti-FLAG M2 antibody (Sigma-Aldrich; Poole, UK); mouse monoclonal anti-phosphotyrosine B5055 (Sigma-Aldrich); mouse monoclonal HA.11 (Covance; Harrogate, UK); rabbit polyclonal anti-caveolin (Santa Cruz Biotechnology; Santa Cruz, CA); histone-1 (Santa Cruz Biotechnology); rabbit polyclonal anti-AcrB (Touze et al., 2004); nickel-nitrilotriacetic acid-HRP (QIAGEN) to detect His-tagged aldolase; rabbit polyclonal anti-Hsp90 (Santa Cruz Biotechnology); rabbit polyclonal anti-N-WASP (gift from Doctor Emma McGhie); mouse monoclonal anti-LAMP H4A3 (Developmental Studies Hybridoma Bank; Iowa City, IA); and rabbit polyclonal anti-calnexin SPA-865 (Stressgen; Ann Arbor, MI).

### Immunoprecipitation

HeLa cells were lysed in buffer N (20 mM Tris-HCl [pH 7.4], 150 mM NaCl, 4 mM MgCl_2_, 0.5% [v/v] CHAPS, 0.2 mM β-mercaptoethanol, and Complete EDTA-free protease inhibitor cocktail [Roche]) and passed through a 22G needle to break up cell debris, and lysates were centrifuged (16,000 *g*, 4°C, 10 min) to isolate detergent-soluble fraction. Proteins were immunoprecipitated with appropriate antibodies immobilized on protein G Sepharose 4 (GE Healthcare). Following incubation at 4°C for 16 hr, precipitated proteins were isolated by centrifugation (2000 *g*, 30 s), washed with buffer N, and then eluted from beads by addition of 1 M glycine (pH 2.0). For immunoprecipitation of phosphorylated VCP, cells were lysed in buffer N supplemented with tyrosine PTPase inhibitors 5 mM Na_3_VO_4_ (vanadate), 5 mM NaF, and 5 mM glycerol-2-phosphate.

### Preparation of Porcine Brain Extract

Fresh pig brains (approximately 650 g, six brains) were homogenized in 1 liter extract buffer using a Waring blender at 4°C. Homogenate was centrifuged (8000 *g*, 30 min, 4°C), and the supernatant (∼800 ml) was filtered through cheesecloth. The filtrate was clarified (12,000 *g*, 4°C, 40 min), and the brain extract was frozen in aliquots at −70°C.

### In Vitro Phosphatase Assays

Recombinant proteins were tyrosine phosphorylated in vitro with constitutively active c-Src kinase according to manufacturer's instructions (Upstate, supplied by Millipore; Watford, UK). Phosphorylated proteins (2 μM) were incubated with serially diluted SptP derivatives in Tris-HCL-buffered saline (TBS) (pH 7.4, 37°C, 5 min), reactions were stopped by 10% (v/v) trichloracetic acid precipitation, and samples were immunoblotted.

## Figures and Tables

**Table 1:** VCP Adaptor Promotion of Sif Formation and *S.* Typhimurium Intracellular Replication

siRNA	Sif-Positive cells (%)	Replication (fold increase)
Control	58.2 ± 1.1	29.6 ± 1.0
Npl4	45.7 ± 1.3	33.4 ± 1.2
Ufd1	38.2 ± 1.2^a^	14.9 ± 2.2^a^
p47	26.7 ± 1.3^a^	7.6 ± 1.2^a^

HeLa cells transfected with control, p47, Npl4, or Ufd1 siRNA were infected with wild-type *S.* Typhimurium, and the number of Sif-positive cells was quantified at 6 hr postinfection after staining with DAPI and antibodies against LAMP1. Intracellular *S.* Typhimurim replication at 8 hr was assayed by colony counts after extracellular bacteria were killed with gentamicin (p < 0.05, ANOVA; n ⩾ 3).

a Significant difference from wild-type.

## Supplemental Data

Document S1. Six Figures

## References

[bib1] Black D.S., Bliska J.B. (1997). Identification of p130Cas as a substrate of *Yersinia* YopH (Yop51), a bacterial protein tyrosine phosphatase that translocates into mammalian cells and targets focal adhesions. EMBO J..

[bib2] Black D.S., Bliska J.B. (2000). The RhoGAP activity of the *Yersinia pseudotuberculosis* cytotoxin YopE is required for antiphagocytic function and virulence. Mol. Microbiol..

[bib3] Brawn L.C., Hayward R.D., Koronakis V. (2007). *Salmonella* SPI1 Effector SipA Persists after Entry and Cooperates with a SPI2 Effector to Regulate Phagosome Maturation and Intracellular Replication. Cell Host Microbe.

[bib4] Brumell J.H., Tang P., Mills S.D., Finlay B.B. (2001). Characterization of *Salmonella*-induced filaments (Sifs) reveals a delayed interaction between *Salmonella*-containing vacuoles and late endocytic compartments. Traffic.

[bib5] Brumell J.H., Tang P., Zaharik M.L., Finlay B.B. (2002). Disruption of the *Salmonella*-containing vacuole leads to increased replication of *Salmonella enterica* serovar Typhimurium in the cytosol of epithelial cells. Infect. Immun..

[bib6] Cain R.J., Hayward R.D., Koronakis V. (2004). The target cell plasma membrane is a critical interface for *Salmonella* cell entry effector-host interplay. Mol. Microbiol..

[bib7] Cossart P., Sansonetti P.J. (2004). Bacterial invasion: the paradigms of enteroinvasive pathogens. Science.

[bib8] Dorer M.S., Kirton D., Bader J.S., Isberg R.R. (2006). RNA interference analysis of *Legionella* in Drosophila cells: exploitation of early secretory apparatus dynamics. PLoS Pathog..

[bib9] Dreveny I., Pye V.E., Beuron F., Briggs L.C., Isaacson R.L., Matthews S.J., McKeown C., Yuan X., Zhang X., Freemont P.S. (2004). p97 and close encounters of every kind: a brief review. Biochem. Soc. Trans..

[bib10] Egerton M., Samelson L.E. (1994). Biochemical characterization of valosin-containing protein, a protein tyrosine kinase substrate in hematopoietic cells. J. Biol. Chem..

[bib11] Flint A.J., Tiganis T., Barford D., Tonks N.K. (1997). Development of “substrate-trapping” mutants to identify physiological substrates of protein tyrosine phosphatases. Proc. Natl. Acad. Sci. USA.

[bib12] Fu Y., Galan J.E. (1998). The *Salmonella typhimurium* tyrosine phosphatase SptP is translocated into host cells and disrupts the actin cytoskeleton. Mol. Microbiol..

[bib13] Fu Y., Galan J.E. (1999). A *salmonella* protein antagonizes Rac-1 and Cdc42 to mediate host-cell recovery after bacterial invasion. Nature.

[bib14] Galan J.E. (2001). *Salmonella* interactions with host cells: type III secretion at work. Annu. Rev. Cell Dev. Biol..

[bib15] Garvis S.G., Beuzon C.R., Holden D.W. (2001). A role for the PhoP/Q regulon in inhibition of fusion between lysosomes and *Salmonella*-containing vacuoles in macrophages. Cell. Microbiol..

[bib16] Hernandez L.D., Hueffer K., Wenk M.R., Galan J.E. (2004). *Salmonella* modulates vesicular traffic by altering phosphoinositide metabolism. Science.

[bib17] Huyer G., Liu S., Kelly J., Moffat J., Payette P., Kennedy B., Tsaprailis G., Gresser M.J., Ramachandran C. (1997). Mechanism of inhibition of protein-tyrosine phosphatases by vanadate and pervanadate. J. Biol. Chem..

[bib18] Kaniga K., Uralil J., Bliska J.B., Galan J.E. (1996). A secreted protein tyrosine phosphatase with modular effector domains in the bacterial pathogen *Salmonella typhimurium*. Mol. Microbiol..

[bib19] Kubori T., Galan J.E. (2003). Temporal regulation of *salmonella* virulence effector function by proteasome-dependent protein degradation. Cell.

[bib20] Kuhle V., Abrahams G.L., Hensel M. (2006). Intracellular *Salmonella enterica* redirect exocytic transport processes in a *Salmonella* pathogenicity island 2-dependent manner. Traffic.

[bib21] Lavoie C., Chevet E., Roy L., Tonks N.K., Fazel A., Posner B.I., Paiement J., Bergeron J.J. (2000). Tyrosine phosphorylation of p97 regulates transitional endoplasmic reticulum assembly in vitro. Proc. Natl. Acad. Sci. USA.

[bib22] Li G., Zhao G., Schindelin H., Lennarz W.J. (2008). Tyrosine phosphorylation of ATPase p97 regulates its activity during ERAD. Biochem. Biophys. Res. Commun..

[bib23] Madsen L., Andersen K.M., Prag S., Moos T., Semple C.A., Seeger M., Hartmann-Petersen R. (2008). Ubxd1 is a novel co-factor of the human p97 ATPase. Int. J. Biochem. Cell Biol..

[bib24] Marcus S.L., Knodler L.A., Finlay B.B. (2002). *Salmonella enterica* serovar Typhimurium effector SigD/SopB is membrane-associated and ubiquitinated inside host cells. Cell. Microbiol..

[bib25] McGhie E.J., Hayward R.D., Koronakis V. (2001). Cooperation between actin-binding proteins of invasive *Salmonella*: SipA potentiates SipC nucleation and bundling of actin. EMBO J..

[bib26] McGhie E.J., Hayward R.D., Koronakis V. (2004). Control of actin turnover by a *salmonella* invasion protein. Mol. Cell.

[bib27] Meresse S., Steele-Mortimer O., Finlay B.B., Gorvel J.P. (1999). The rab7 GTPase controls the maturation of *Salmonella typhimurium*-containing vacuoles in HeLa cells. EMBO J..

[bib28] Murli S., Watson R.O., Galan J.E. (2001). Role of tyrosine kinases and the tyrosine phosphatase SptP in the interaction of *Salmonella* with host cells. Cell. Microbiol..

[bib29] Patel J.C., Galan J.E. (2006). Differential activation and function of Rho GTPases during *Salmonella*-host cell interactions. J. Cell Biol..

[bib30] Perrin A.J., Jiang X., Birmingham C.L., So N.S., Brumell J.H. (2004). Recognition of bacteria in the cytosol of Mammalian cells by the ubiquitin system. Curr. Biol..

[bib31] Pye V.E., Beuron F., Keetch C.A., McKeown C., Robinson C.V., Meyer H.H., Zhang X., Freemont P.S. (2007). Structural insights into the p97-Ufd1-Npl4 complex. Proc. Natl. Acad. Sci. USA.

[bib32] Rabouille C., Kondo H., Newman R., Hui N., Freemont P., Warren G. (1998). Syntaxin 5 is a common component of the NSF- and p97-mediated reassembly pathways of Golgi cisternae from mitotic Golgi fragments in vitro. Cell.

[bib33] Salcedo S.P., Holden D.W. (2003). SseG, a virulence protein that targets *Salmonella* to the Golgi network. EMBO J..

[bib34] Schuberth C., Buchberger A. (2008). UBX domain proteins: major regulators of the AAA ATPase Cdc48/p97. Cell. Mol. Life Sci..

[bib35] Stebbins C.E., Galan J.E. (2000). Modulation of host signaling by a bacterial mimic: structure of the *Salmonella* effector SptP bound to Rac1. Mol. Cell.

[bib36] Steele-Mortimer O. (2008). The *Salmonella*-containing vacuole: Moving with the times. Curr. Opin. Microbiol..

[bib37] Steele-Mortimer O., Meresse S., Gorvel J.P., Toh B.H., Finlay B.B. (1999). Biogenesis of *Salmonella typhimurium*-containing vacuoles in epithelial cells involves interactions with the early endocytic pathway. Cell. Microbiol..

[bib38] Steele-Mortimer O., Brumell J.H., Knodler L.A., Meresse S., Lopez A., Finlay B.B. (2002). The invasion-associated type III secretion system of *Salmonella enterica* serovar Typhimurium is necessary for intracellular proliferation and vacuole biogenesis in epithelial cells. Cell. Microbiol..

[bib39] Tiganis T., Bennett A.M. (2007). Protein tyrosine phosphatase function: the substrate perspective. Biochem. J..

[bib40] Touze T., Eswaran J., Bokma E., Koronakis E., Hughes C., Koronakis V. (2004). Interactions underlying assembly of the Escherichia coli AcrAB-TolC multidrug efflux system. Mol. Microbiol..

[bib41] Wojcik C., Yano M., DeMartino G.N. (2004). RNA interference of valosin-containing protein (VCP/p97) reveals multiple cellular roles linked to ubiquitin/proteasome-dependent proteolysis. J. Cell Sci..

[bib42] Ye Y. (2006). Diverse functions with a common regulator: ubiquitin takes command of an AAA ATPase. J. Struct. Biol..

[bib43] Yeung H.O., Kloppsteck P., Niwa H., Isaacson R.L., Matthews S., Zhang X., Freemont P.S. (2008). Insights into adaptor binding to the AAA protein p97. Biochem. Soc. Trans..

[bib44] Zhao G., Zhou X., Wang L., Li G., Schindelin H., Lennarz W.J. (2007). Studies on peptide:N-glycanase-p97 interaction suggest that p97 phosphorylation modulates endoplasmic reticulum-associated degradation. Proc. Natl. Acad. Sci. USA.

