# Supplementary material for: The Salmonella Effector SptP Dephosphorylates Host AAA+ ATPase VCP to Promote Development of its Intracellular Replicative Niche
Source: Cell Host Microbe. 2009 Mar 19;5(3-3):225–33. doi: 10.1016/j.chom.2009.01.010 (PMC2724103; doi:10.1016/j.chom.2009.01.010)
Supplement: Document S1. Six Figures [file mmc1.pdf]

Supplemental Data

Cell Host & Microbe, *Volume 5*

The Salmonella Effector SptP Dephosphorylates Host AAA+ ATPase VCP to Promote Development of its Intracellular Replicative Niche

Daniel Humphreys, Peter J. Hume, and Vassilis Koronakis

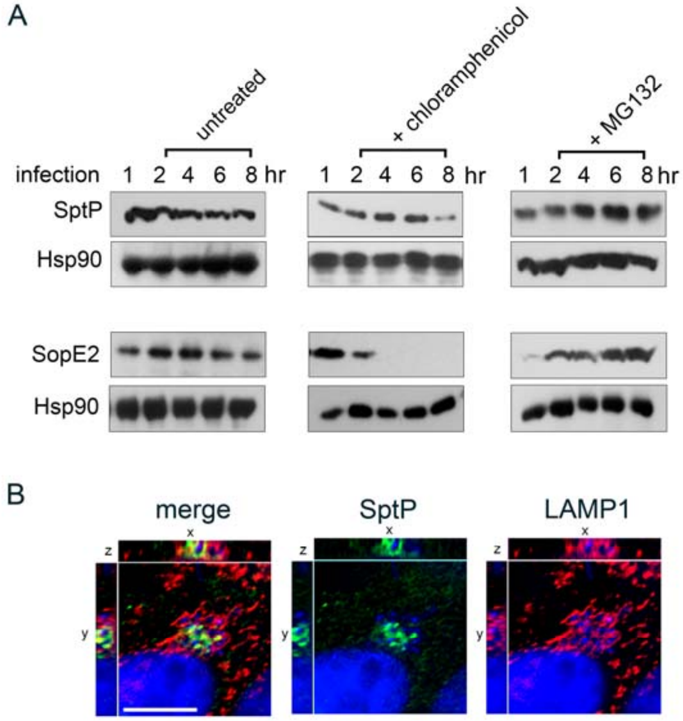

Figure S1. Persistence and Localization of SptP Following Pathogen Entry into Host Cells

(A) Immunoblots showing the persistence of SptP and SopE2 after cell infection. HeLa cells were infected with *S.* Typhimurium expressing SptP^{FLAG} or SopE2^{FLAG}, and at 1 hr the cells were left untreated, treated with either the proteasome inhibitor MG132 or the bacterial protein synthesis inhibitor chloramphenicol. Cells were lysed with detergent and effectors immunoprecipitated with antibodies against FLAG immobilized on protein-G Sepharose. Immunoprecipitates containing bound proteins were assayed for presence of SptP and SopE2 with antibodies against FLAG. Unbound supernatants were immunoblotted for Hsp90 as control. SptP persists inside host cells for at least 8 hr (top left). When untreated infected cells were compared to those incubated from 1 hr with chloramphenicol, the levels of SptP were unaffected and the effector still persisted for 8 hr showing that SptP is delivered during the entry process and remains inside host cells due to its intrinsic stability (top middle). When compared to untreated infected cells those incubated from 1 hr with MG132 revealed no significant differences in the levels of SptP confirming that it is only delivered early during cell infection (top right). SopE2 also persists for at least 8 hr (bottom left). In contrast to SptP, infected cells incubated with chloramphenicol led to the degradation of SopE2 within 1 hr (bottom middle) while incubation with MG132 resulted in increased levels of SopE2 (bottom right). Therefore, SopE2 persists through its continual delivery by intracellular *Salmonella* to counteract its rapid degradation in a proteasome-dependent manner. Collectively, these data show that SptP is stable inside host cells while SopE2 is not.

(B) Localization of both SptP and LAMP1 during HeLa cell infection by WT *S.* Typhimurium. At 6 hr postinfection, cells were stained with DAPI (host nuclei and bacteria; blue) and antibodies against LAMP1 (red) and FLAG (SptP; green). Immunofluorescence images are shown in XY (large panels), YZ-cross-section (left) and XZ-cross-section (top). The Z sections were acquired each 0.2 μm and deconvolved using Volocity software (Improvision). Scale bar, 7 μm.

A

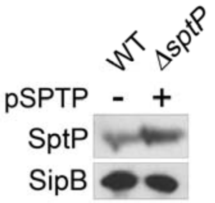

B

S. Typhimurium strain	Sif + ve cells (%)	replication (fold increase)	LAMP1 + ve bacteria (%)
wild-type	54.6 ± 1.1	14.3 ± 1.4	89.4 ± 1.2
ΔsptP	26.4 ± 1.1*	4.2 ± 2.6*	92.4 ± 1.1
ΔsptP pSPTP	49.2 ± 1.1	35.2 ± 1.4*	88.1 ± 1.8
ΔsptP pR209A	47.4 ± 1.2	28.2 ± 2.8*	89.1 ± 2.7
ΔsptP pD441A	15.5 ± 1.4*	6.4 ± 1.3*	90.3 ± 2.2

C

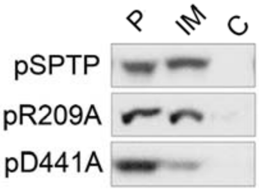

Figure S2. Influence of SptP on Intracellular Replication and Sif Formation

(A) Immunoblot showing levels of translocated SptP. HeLa cells infected (1 hr) with WT *S.* Typhimurium or the *sptP* null mutant (*ΔsptP*) expressing plasmid-encoded SptP (pSPTP) were lysed in buffer N containing detergent to extract soluble translocated proteins. Samples were immunoblotted with antibodies against SipB and FLAG (SptP).

(B) Table showing SptP promotion of Sif formation and *S.* Typhimurium intracellular replication during cell infection. HeLa cells were infected in parallel with the indicated strains and the number of Sif positive (+ve) cells quantified at 6 hr and the number of LAMP1-positive SCVs quantified at 8 hr postinfection after staining with DAPI and antibodies against LAMP1. Intracellular *S.* Typhimurium replication at 8 hr was assayed by colony counts (geometric mean ± 95% confidence intervals) after extracellular bacteria were killed with gentamicin. Asterisks indicate a significant difference from WT ($p < 0.05$, ANOVA. $n \geq 3$).

(C) Immunoblot showing subcellular localization of WT $\text{SptP}^{\text{FLAG}}$ and mutant variants $\text{SptP}^{\text{D441A-FLAG}}$ and $\text{SptP}^{\text{R209A-FLAG}}$ during cell infection. HeLa cells infected (8 hr) with *S.* Typhimurium *ΔsptP* expressing FLAG-tagged SptP variants from indicated plasmids were mechanically fractionated into the pellet (P), internal membranes (IM), and host cytoplasm (C). Samples were immunoblotted with antibodies against FLAG.

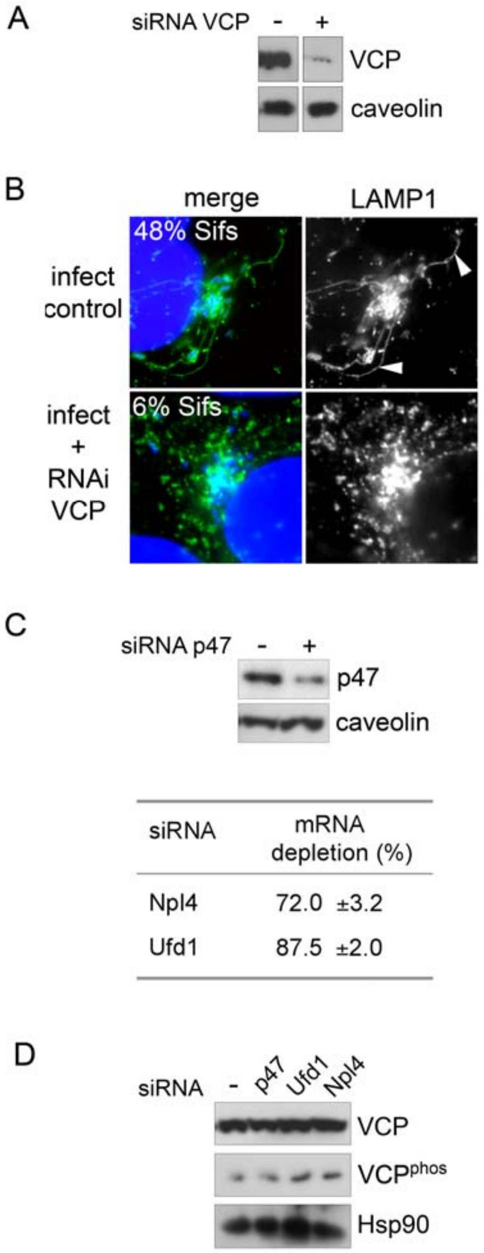

siRNA	mRNA depletion (%)
Npl4	72.0 ±3.2
Ufd1	87.5 ±2.0

Figure S3. Influence of VCP on Cell Infection by *Salmonella*

(A) Immunoblot showing cellular levels of VCP after VCP siRNA transfection. Cell lysates from HeLa cells 72 hr after treatment with VCP siRNA (+) or control siRNA (-) were assayed for VCP by immunoblotting using antibodies against VCP and caveolin as a loading control.

(B) Inhibition of Sif formation in VCP siRNA transfected cells. HeLa cells treated with VCP siRNA or control siRNA were infected with WT *S.* Typhimurium. At 6 hr postinfection, cells were stained with DAPI and antibodies against LAMP1 to visualise Sifs (arrowed). The percentage of Sif positive cells is indicated (±3%).

(C) Immunoblot (upper) showing cellular levels of p47 after p47 siRNA transfection as (A) using antibodies against p47 and caveolin as a loading control. Table (lower) showing levels of Npl4 and Ufd1 mRNA depletion after siRNA transfection. Levels of cDNA were quantified from HeLa cells 72 hr after treatment with either Npl4 siRNA or Ufd1 siRNA by qRT-PCR.

(D) Immunoblot showing cellular levels of VCP and its phosphorylation status after p47, Ufd1, and Npl4 siRNA transfection. Cell lysates from HeLa cells (representing 50% of total cell mass) 72 hr after treatment with control (-), p47, Ufd1, or Npl4 siRNA were assayed for VCP by immunoblotting using antibodies against VCP and Hsp90 as a loading control. VCP was immunoprecipitated from the remaining HeLa cells (50%) using antibodies against VCP immobilised on protein-G Sepharose. Phosphorylation status of immunoprecipitated VCP was assessed by immunoblotting with antibodies against phosphotyrosine (VCP^{phos}).

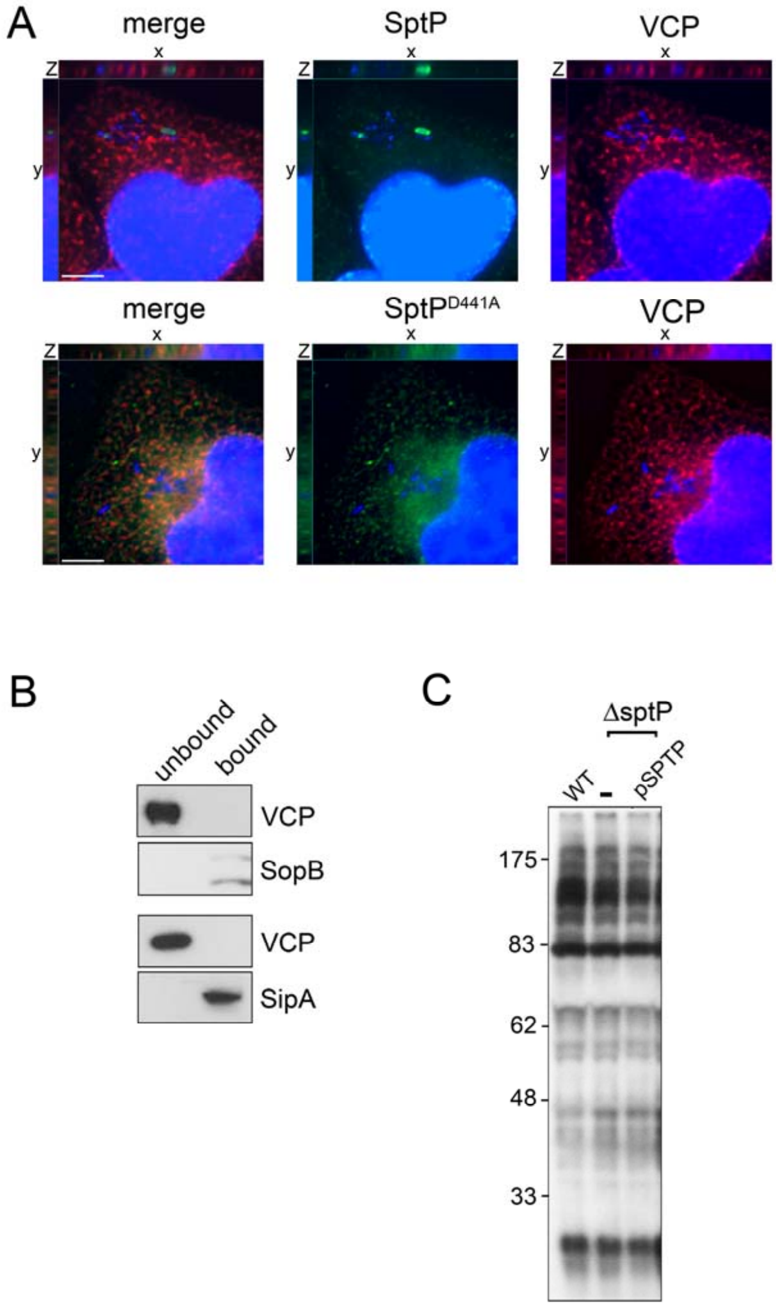

Figure S4. Interaction of VCP with *Salmonella* Effectors

(A) Localization of SptP^{D441A} and VCP during HeLa cell infection. At 6 hr postinfection with either *S.* Typhimurium *ΔsptP* expressing plasmid-encoded FLAG-tagged SptP or SptP^{D441A}, cells were stained with DAPI (host nuclei and bacteria; blue) and antibodies against VCP (red) and FLAG (SptP; green). Immunofluorscence images are shown in XY (large panels), YZ-cross-section (left) and XZ-cross-section (top). The Z sections were acquired each 0.2 μm and deconvolved using Volocity software (Improvision). Scale bar 5 μm. Top panel; SptP accumulated around intracellular bacteria (SptP) and does not colocalize with VCP (merge), which was distributed throughout the cell (VCP). Bottom panel; the substrate-trap SptP^{D441A} was absent from SCVs and was enriched at the perinuclear region of the cell (SptP^{D441A}; Figure S2C). VCP was evident throughout the cell and was enriched on membrane-like structures (VCP), some of which colocalized with SptP^{D441A} (merge).

(B) Immunoblot showing SopB and SipA interaction with VCP during cell infection. HeLa cells infected (4 hr) with *S.* Typhimurium expressing SopB^{FLAG} or SipA^{FLAG} were lysed with detergent and effectors immunoprecipitated with antibodies against FLAG immobilized on protein-G Sepharose. Immunoprecipitates containing bound proteins and unbound supernatants were assayed for presence of SopB, SipA, and VCP with antibodies against FLAG and VCP.

(C) Total phosphotyrosine in host cell lysates. Lysates of HeLa cells infected (4 hr) with WT, *ΔsptP*, and *ΔsptP* pSPTP *S.* Typhimurium were immunoblotted with antibodies against phosphotyrosine.

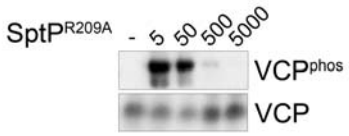

Figure S5. PTPase Activity of GAP-Defective SptP^{R209A} towards VCP^{WT}

In vitro phosphorylated VCP^{WT} (2 μM) were incubated with picomolar concentrations of SptP^{R209A}. Phosphorylation was assessed by immunoblotting samples with antibodies against phosphotyrosine (VCP^{phos}) and VCP as control.

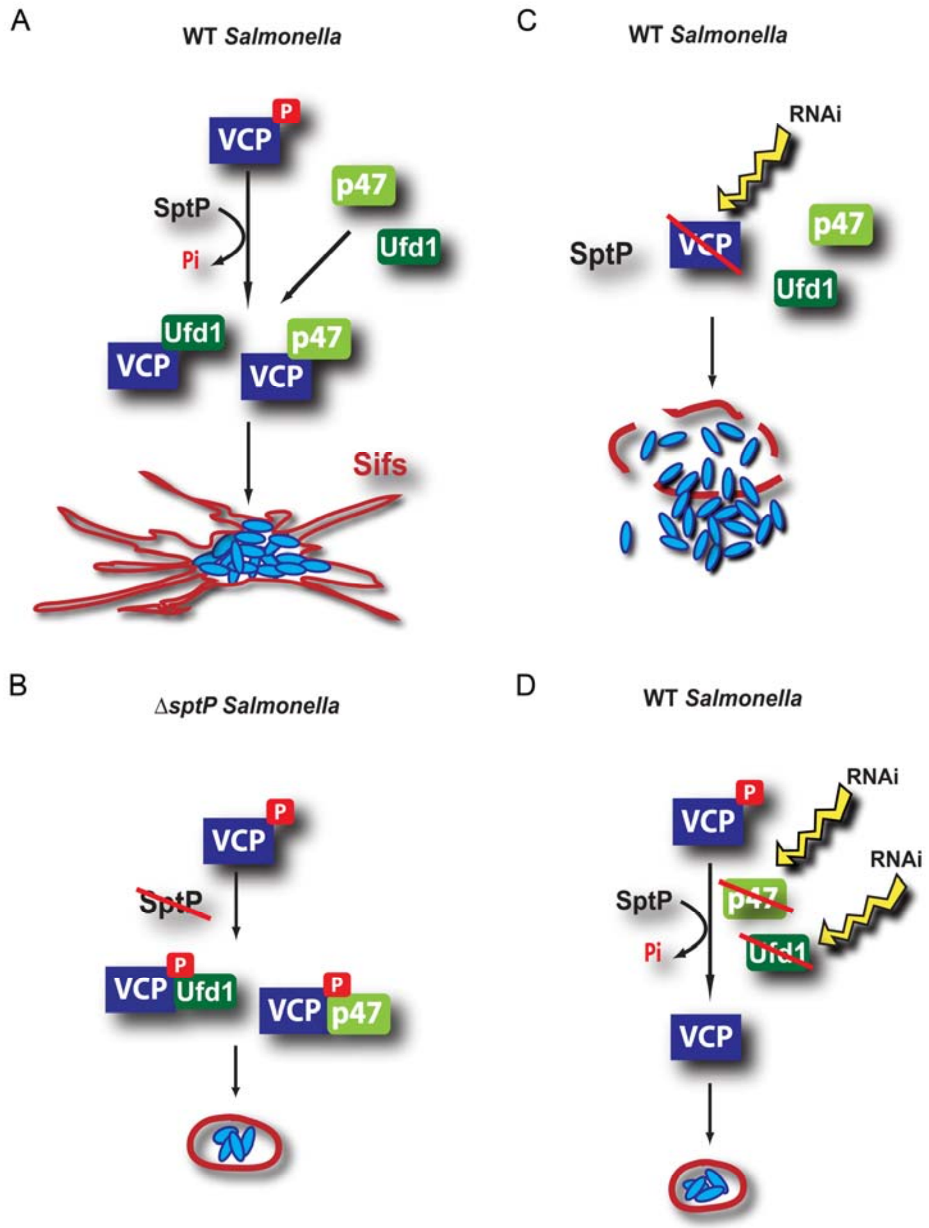

Figure S6. Model for SptP Promotion of Intracellular Replication

(A) SptP PTPase activity promotes intracellular replication. SptP-dependent dephosphorylation of VCP allows it to promote Sif formation and intracellular replication through the adaptors p47 and Ufd1.

(B) *sptP* null *Salmonella* are retarded for intracellular replication. Internalized Δ*sptP Salmonella* reside in SCVs but VCP is not dephosphorylated. This prevents association with p47 and Ufd1 and thus impairs the ability of VCP to promote Sif formation and intracellular replication.

(C) VCP is necessary for multiple pathways that promote intracellular replication. Internalized *Salmonella* reside in SCVs but RNAi-depletion of VCP impairs the p47 and Ufd1 (and possibly other) pathways. This leads to loss of SCV integrity and hyper-replication of *Salmonella* in the HeLa cell cytosol.

(D) VCP adaptors p47 and Ufd1 promote intracellular replication. Internalised *Salmonella* reside in SCVs, VCP is dephosphorylated but RNAi-depletion of p47 and Ufd1 prevents VCP promoting Sif formation and intracellular replication.
